# Impact of Genetic Variation on Human CaMKK2 Regulation by Ca^2+^-Calmodulin and Multisite Phosphorylation

**DOI:** 10.1038/srep43264

**Published:** 2017-02-23

**Authors:** Matthew T. O’Brien, Jonathan S. Oakhill, Naomi X. Y. Ling, Christopher G. Langendorf, Ashfaqul Hoque, Toby A. Dite, Anthony R. Means, Bruce E. Kemp, John W. Scott

**Affiliations:** 1 St Vincent’s Institute and Department of Medicine, University of Melbourne, 41 Victoria Parade, Fitzroy, 3065, Australia; 2 Mary MacKillop Institute for Health Research, Australian Catholic University, 215 Spring Street, Melbourne, 3000, Australia; 3 Department of Molecular and Cellular Biology, Baylor College of Medicine, Houston, TX, 77030, USA

## Abstract

The Ca^2+^-calmodulin dependent protein kinase kinase-2 (CaMKK2) is a key regulator of neuronal function and whole-body energy metabolism. Elevated CaMKK2 activity is strongly associated with prostate and hepatic cancers, whereas reduced CaMKK2 activity has been linked to schizophrenia and bipolar disease in humans. Here we report the functional effects of nine rare-variant point mutations that were detected in large-scale human genetic studies and cancer tissues, all of which occur close to two regulatory phosphorylation sites and the catalytic site on human CaMKK2. Four mutations (G87R, R139W, R142W and E268K) cause a marked decrease in Ca^2+^-independent autonomous activity, however S137L and P138S mutants displayed increased autonomous and Ca^2+^-CaM stimulated activities. Furthermore, the G87R mutant is defective in Thr85-autophosphorylation dependent autonomous activity, whereas the A329T mutation rendered CaMKK2 virtually insensitive to Ca^2+^-CaM stimulation. The G87R and R139W mutants behave as dominant-negative inhibitors of CaMKK2 signaling in cells as they block phosphorylation of the downstream substrate AMP-activated protein kinase (AMPK) in response to ionomycin. Our study provides insight into functionally disruptive, rare-variant mutations in human CaMKK2, which have the potential to influence risk and burden of disease associated with aberrant CaMKK2 activity in human populations carrying these variants.

The Ca^2+^-calmodulin (CaM) dependent protein kinase kinase-2 (CaMKK2) is the central component of a signaling cascade that activates Ca^2+^-CaM dependent protein kinase-I (CaMK-I), Ca^2+^-CaM dependent protein kinase-IV (CaMK-IV), the AMP-activated protein kinase (AMPK), and the histone deacetylase Sirt1 signaling pathways to regulate a variety of physiological processes including emotional behaviour, endothelial function, and whole-body energy metabolism^1^. Similar to other CaMK family members, CaMKK2 is composed of divergent N- and C-terminal sequences that encompass a mid-molecule kinase domain and a regulatory module comprising overlapping autoinhibitory and CaM binding sequences^2^. Under resting conditions, the autoinhibitory sequence is thought to obstruct the active site by an intra-steric mechanism, which is relieved by Ca^2+^-CaM binding^3^. When bound to Ca^2+^-CaM, CaMKK2 undergoes autophosphorylation at Thr85, which generates Ca^2+^-independent autonomous activity by maintaining CaMKK2 in the activated state after removal of the Ca^2+^ stimulus^4^.

The Ca^2+^-CaM dependence of CaMKK2 is unique among the CaM kinase subfamily, being contingent upon sequential phosphorylation of Ser129, Ser133 and Ser137 located within a regulatory sequence N-terminal to the catalytic domain^5^. Phosphorylation of Ser137 by proline-directed kinases primes CaMKK2 for subsequent phosphorylation on Ser133 and Ser129 by glycogen synthase kinase-3 (GSK3). All three sites are constitutively phosphorylated in mammalian cells under resting conditions, which renders CaMKK2 fully Ca^2+^-CaM dependent and suppresses autonomous activity, whereas dephosphorylation of Ser129/Ser133/Ser137 increases autonomous activity^5^.

Elevated CaMKK2 activity is strongly associated with a number of disease states in humans. CaMKK2 expression is up-regulated in hepato-cellular carcinoma (HCC) and negatively correlates with HCC patient survival^6^. Furthermore, CaMKK2 expression is markedly increased in a carcinogen-induced HCC mouse model and pharmacological inhibition of CaMKK2 with STO-609 regresses hepatic tumor burden. CaMKK2 is also highly expressed in prostate cancer tumors and is an important androgen receptor-regulated gene^7^. Expression of CaMKK2 increases during the transition from benign prostate tumor growth to prostate cancer, and is thought to be a key driver in the development of castration-resistant prostate tumors^8^. On the other hand, behavioural disorders such as schizophrenia and bipolar disease are strongly linked to decreased CaMKK2 activity. An intronic mutation that reduces *CaMKK2* expression (rs1063843), as well as a mutation that occurs at the regulatory Thr85 autophosphorylation site (T85S, rs3817190) have been linked with schizophrenia and bipolar disorder in humans, respectively^9^,^10^,^11^. Strikingly, autophosphorylation of Ser85 in the T85S mutant fails to increase autonomous activity and instead causes a suppression of CaMKK2 activity^4^. Consistent with the human mutations, CaMKK2 null mice modeling loss of function exhibit anxiety and manic-like behavioral disturbances^4^. CaMKK2 expression has been reported to be reduced in human glioblastomas, as well as in cell lines derived from glioblastoma patients compared with normal brain tissue^12^. However, other studies have reported that CaMKK2 expression is increased in high-grade human glioma samples, and correlates with poor prognosis^13^.

Nine rare-variant point mutations that occur in close proximity to regulatory phosphorylation sites and the catalytic site on human CaMKK2 have been detected in large-scale genetic studies^14^,^15^, as well as in several cancer cell lines and tissues derived from cancer patients^16^,^17^,^18^,^19^,^20^. Rare-variant mutations are considered to be an important component in genetic susceptibility to common human diseases, and are more likely to be functionally disruptive and hence have larger effects on disease risk than common variants^21^,^22^. This prompted us to analyze the functional effects of human CaMKK2 rare-variant mutations on enzyme activity and regulation by Ca^2+^-CaM and multisite phosphorylation.

## Results

### The G87R mutation prevents Thr85 autophosphorylation and decreases autonomous activity

**Figure 1:**
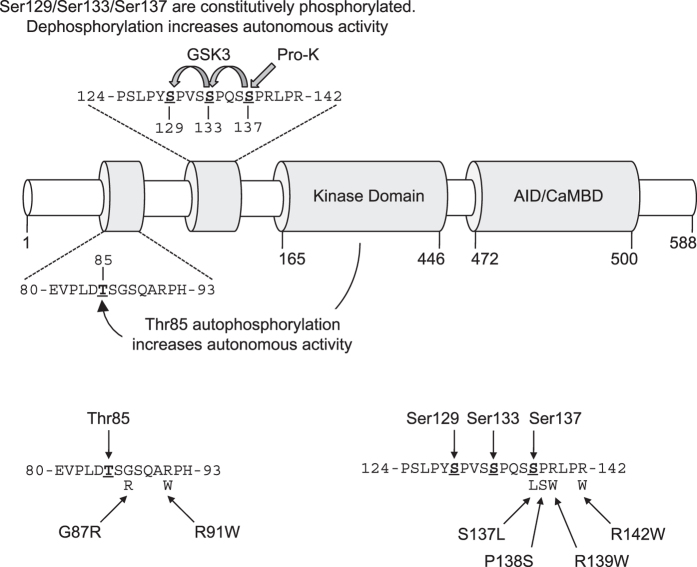
Rare variant point mutations within human CaMKK2 located close to regulatory phosphorylation sites. Linear schematic of CaMKK2 domain structure showing the regulatory phosphorylation sites, kinase domain and overlapping autoinhibitory (AID) and calmodulin-binding (CaMBD) domains. Amino acid sequences (residues 80–93 and residues 124–142) show the location of rare variant point mutations (G87R, R91W, S137L, P138S, R139W, R142W) with respect to the regulatory Thr85 autophosphorylation and Ser129/Ser133/Ser137 phosphorylation sites.

**Figure 2:**
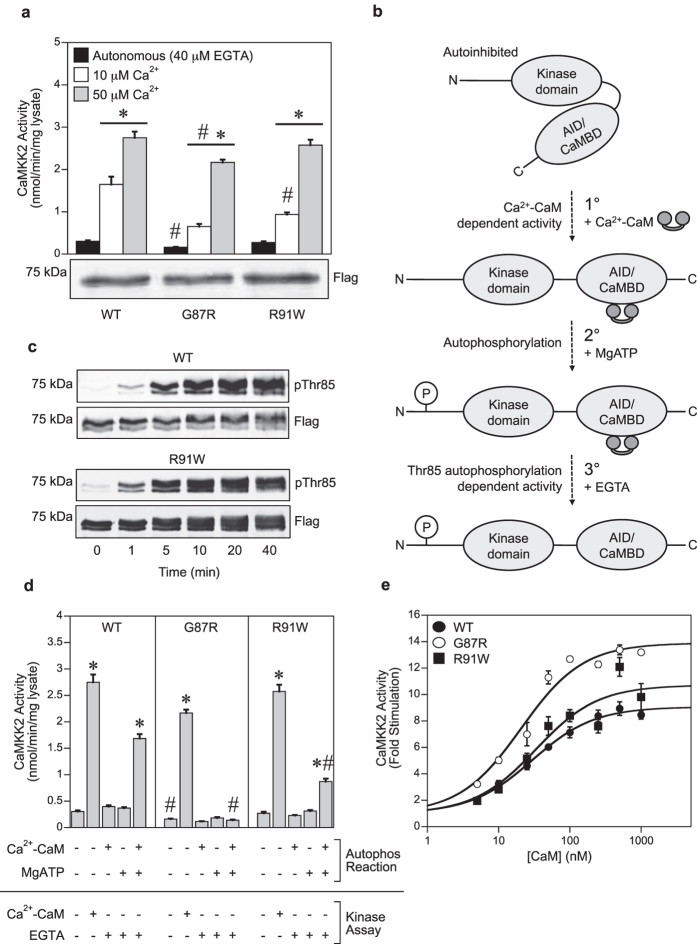
The G87R mutant is defective in Thr85-autophosphorylation dependent autonomous activity. (**a**) Autonomous and Ca^2+^-CaM stimulated activities of WT and mutant CaMKK2 determined in the presence of 40 μM EGTA, 1 μM CaM, 200 μM ATP and either 10 μM or 50 μM Ca^2+^. CaMKK2 activity was measured using the CaMKKtide peptide substrate assay and the data presented as the mean ± SEM; n = 4. The lower panel shows expression levels of WT CaMKK2 and the mutants as determined by immunoblot using a rabbit anti-Flag antibody. (**b**) Schematic showing the mechanism by which CaMKK2 generates increased autonomous activity via Thr85 autophosphorylation. (**c**) Immunoblot analysis of Thr85 autophosphorylation in WT and mutant CaMKK2 determined at various time points (0–40 min) after incubation with 50 μM Ca^2+^, 1 μM CaM and 200 μM MgATP. Thr85 autophosphorylation and total CaMKK2 protein were detected (shown on a cropped representative immunoblot) using rabbit pThr85 and mouse anti-Flag antibodies, respectively. (**d**) Effect of G87R and R91W mutations on generating increased autonomous activity via autophosphorylation. The autophosphorylation reaction was performed using CaMKK2 immobilized on Flag-agarose beads in the presence and absence of 50 μM Ca^2+^, 1 μM CaM and 200 μM MgATP for 40 min, after which the reaction was quenched by extensively washing the beads to remove the autophosphorylation reaction components. Activity was then measured in the presence and absence of 50 μM Ca^2+^, 1 μM CaM and 1 mM EGTA as indicated using the CaMKKtide peptide substrate kinase assay. Data are presented as mean ± SEM; n = 4. (**e**) Activation of WT and mutant CaMKK2 measured over a range of CaM concentrations (0–1000 nM) in the presence of 50 μM Ca^2+^. CaMKK2 activity was measured using the CaMKKtide peptide substrate assay and data were fitted to the equation: Activity = Basal + ((((Fold Stimulation × Basal) − Basal) × [CaM])/(A_0.5_ + [CaM])), where A_0.5_ is the concentration of CaM giving half-maximal stimulation. Data are presented as mean ± SEM; n = 4. Statistical analysis was performed by two-way ANOVA. *p < 0.001, vs the control within the same group; #p < 0.001 vs WT within the same treatment.

Two point mutations (G87R, rs200654946; R91W, rs200162363) that occur adjacent to the Thr85 autophosphorylation site (Fig. 1) have been detected in a number of large-scale population studies of human genetic variation^15^,^23^,^24^. The G87R mutation has also been detected in non-Neurofibromatosis Type 2 (NF2) meningiomas^17^, and the R91W mutation in the LOVO cell line derived from human colorectal adenocarcinoma^19^,^25^,^26^. Using recombinant human CaMKK2 immuno-precipitated from COS7 cells, we first measured Ca^2+^-independent autonomous activity and Ca^2+^-CaM stimulated activity of WT, G87R and R91W mutant CaMKK2. Autonomous activity was first demonstrated in CaMKII, and is defined as the kinase activity that is measured when the Ca^2+^ stimulus (but not CaM) is removed with the chelator EGTA^27^,^28^. CaMKK2 autonomous activity was measured in the presence of 40 μM EGTA, which we determined by titration in order to chelate contaminating Ca^2+^ in the CaM preparation (Supplementary Fig. 1). Ca^2+^-CaM stimulated activity was determined in the presence of either 50 μM Ca^2+^ (which is a supra-physiological Ca^2+^ concentration but gives maximal stimulation of CaMKK2 *in vitro*^4^) or 10 μM Ca^2+^ that is within the physiological range^29^. As shown in Fig. 2a, we found that the G87R mutant had substantially reduced autonomous activity as well as lower Ca^2+^-CaM stimulated activity at both Ca^2+^ concentrations compared with WT CaMKK2. The R91W mutant had similar autonomous activity and Ca^2+^-CaM stimulated activity at 50 μM Ca^2+^ as WT CaMKK2, however kinase activity at 10 μM Ca^2+^ was significantly lower. Immunoblot analysis revealed similar levels of expression of WT CaMKK2 and the G87R and R91W mutants, indicating that the changes in activity were not due to differences in the level of expression (Fig. 2a). We observed that recombinant CaMKK2 migrates as an apparent doublet on SDS-PAGE (Fig. 2a,c). This is due to translation from Met49, as a M49L mutation generated a single species corresponding to full length WT CaMKK2 that migrated with the upper band (Supplementary Fig. 2a). Similarly, a CaMKK2 mutant lacking residues 1–48 (Δ1–48) migrated with the lower band in the doublet that corresponded with the alternative translated CaMKK2 initiated at Met49. The CaMKK2 (48–588) isoform has a similar post-translational profile as CaMKK2 (1–588) as shown by whole-protein TOF-MS (Supplementary Fig. 3a,b).

We next examined whether either of the R91W or G87R mutations alter the ability of CaMKK2 to autophosphorylate Thr85, and therefore generate increased autonomous activity (Fig. 2b). Similar to WT CaMKK2, the R91W mutant was capable of undergoing Thr85 autophosphorylation in the presence of Ca^2+^-CaM and MgATP (Fig. 2c), whereas Thr85 autophosphorylation was virtually abolished in the G87R mutant (Supplementary Fig. 4; mass spectrometry was used to detect Thr85 autophosphorylation in the G87R mutant because this mutation disrupts the pThr85 phospho-specific antibody recognition). Autophosphorylation of WT CaMKK2 in the presence of Ca^2+^-CaM and MgATP yielded a 5-fold increase in activity that persisted in the presence of EGTA (Fig. 2d). However, the ability to increase autonomous activity via Thr85 autophosphorylation was markedly reduced in the R91W mutant, and completely lost in the G87R mutant (Fig. 2d).

Although the G87R and R91W mutations are distantly located from the CaM-binding site (residues 475–500) in the primary sequence of CaMKK2 (Fig. 1), intra-molecular crosstalk between these two regulatory regions is evident since initiation of Thr85 autophosphorylation is Ca^2+^-CaM dependent. We therefore determined CaM sensitivity and dependence of the G87R and R91W mutants, by measuring Ca^2+^-CaM stimulation over a range of CaM concentrations (0–1000 nM) at a fixed concentration (50 μM) of Ca^2+^. Neither mutation had a significant effect on the concentration of CaM required for half-maximal stimulation, indicating that CaM sensitivity was unaltered (Fig. 2e; Table 1). However, the G87R mutant was more dependent on CaM to achieve maximal activation (8.64-fold stimulation for WT versus 13.2-fold for G87R) (Fig. 2e; Table 1).

### The R139W mutation prevents phosphorylation of Ser129 and decreases autonomous and Ca^2+^-CaM stimulated activity

**Figure 3:**
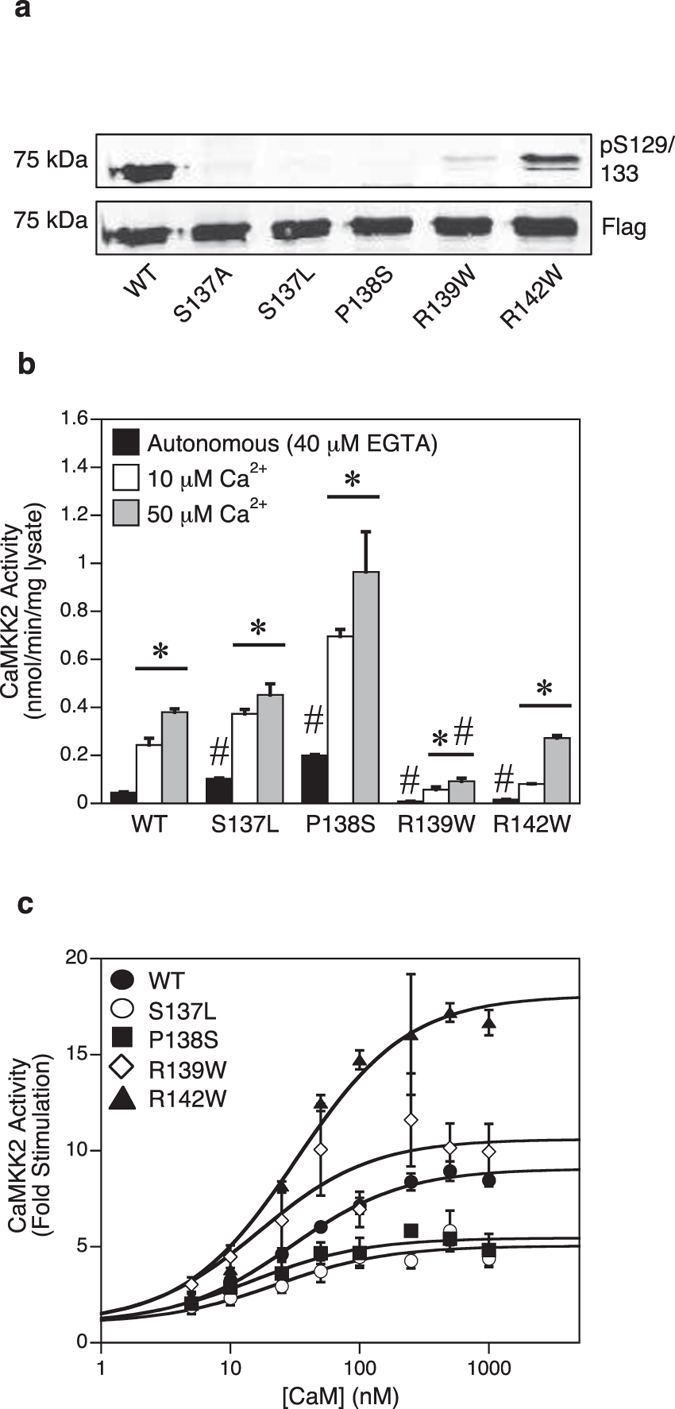
Mutations at or in proximity to Ser137 can increase or decrease autonomous and Ca^2+^-CaM stimulated activity. (**a**) Immunoblot analysis of Ser129 phosphorylation in WT and mutant CaMKK2. Phosphorylation of Ser129 and total CaMKK2 protein were determined (shown on a cropped representative immunoblot) using mouse phosphoserine and rabbit anti-Flag antibodies. The S137A mutant, which we showed previously prevents sequential phosphorylation on the Ser129/Ser133 sites^5^, was included as a positive control. (**b**) Autonomous and Ca^2+^-CaM stimulated activities of WT and mutant CaMKK2 determined in the presence of 40 μM EGTA, 1 μM CaM, 200 μM ATP and either 10 μM or 50 μM Ca^2+^. CaMKK2 activity was measured using the CaMKKtide peptide substrate assay and the data presented as the mean ± SEM; n = 4–8. (**c**) Kinase activity of WT and mutant CaMKK2 measured over a range of CaM concentrations (0–1000 nM) in the presence of 50 μM Ca^2+^. CaMKK2 activity was measured using the CaMKKtide peptide substrate assay and data were fitted to the equation: Activity = Basal + ((((Fold Stimulation × Basal) − Basal) × [CaM])/(A_0.5_ + [CaM])), where A_0.5_ is the concentration of CaM giving half-maximal stimulation. Data are presented as mean ± SEM; n = 4–8. Statistical analysis was performed by two-way ANOVA. *p < 0.01, vs the control within the same group; ^#^p < 0.001 vs WT within the same treatment.

We next examined four point mutations (S137L, rs201145728; P138S, rs199839786; R139W, rs2020448546; R142W, rs201263450) that were detected in two human genetic variation studies^15^,^24^, which occur at or in close proximity to the regulatory Ser137 phosphorylation site on human CaMKK2 (Fig. 1). Since dephosphorylation of Ser129/Ser133/Ser137 is an important mechanism for increasing autonomous activity in the absence of Ca^2+^-CaM^5^, we initially investigated whether the mutations alter the phosphorylation profile of human CaMKK2 expressed in mammalian COS7 cells. We performed immunoblot analysis on WT and mutant CaMKK2 using a phospho-Ser129 specific antibody (Fig. 3a). WT CaMKK2 was phosphorylated on Ser129 as expected, however there was near complete loss of phosphorylation in the S137L, P138S and R139W mutants, and a partial loss of phosphorylation in the R142W mutant. We next measured autonomous activity (40 μM EGTA), and Ca^2+^-CaM stimulated activity in the presence of either 10 μM or 50 μM Ca^2+^. Consistent with loss of phosphorylation of Ser129/Ser133/Ser137, both the S137L and P138S mutants exhibited a 2.3- and 4.5-fold increase in autonomous activity relative to WT CaMKK2, respectively (Fig. 3b). Moreover, the Ca^2+^-CaM stimulated activity of the P138S mutant was higher than WT CaMKK2. Surprisingly, the R139W and R142W mutants had substantially reduced autonomous activities compared to WT CaMKK2 despite both mutants showing decreased Ser129/Ser133/Ser137 phosphorylation, and also had markedly reduced Ca^2+^-CaM stimulated activities. All the mutants were expressed at similar levels to WT CaMKK2 (Fig. 3a), indicating that the alterations in kinase activity were due to intrinsic changes in enzyme function.

We next measured CaM sensitivity and dependence of the mutants, and found that the P138S and R139W mutations rendered CaMKK2 more sensitive to stimulation with Ca^2+^-CaM, as shown by the lower concentration of CaM required for half-maximal stimulation compared with WT CaMKK2 (Fig. 3c; Table 1). The R142W mutant was more CaM-dependent (8.64-fold stimulation for WT versus 18.1-fold for R142W), whereas the S137L and P138S mutants showed reduced CaM-dependence (Fig. 3c; Table 1).

### The A329T mutant is insensitive to Ca^2+^-CaM stimulation

**Figure 4:**
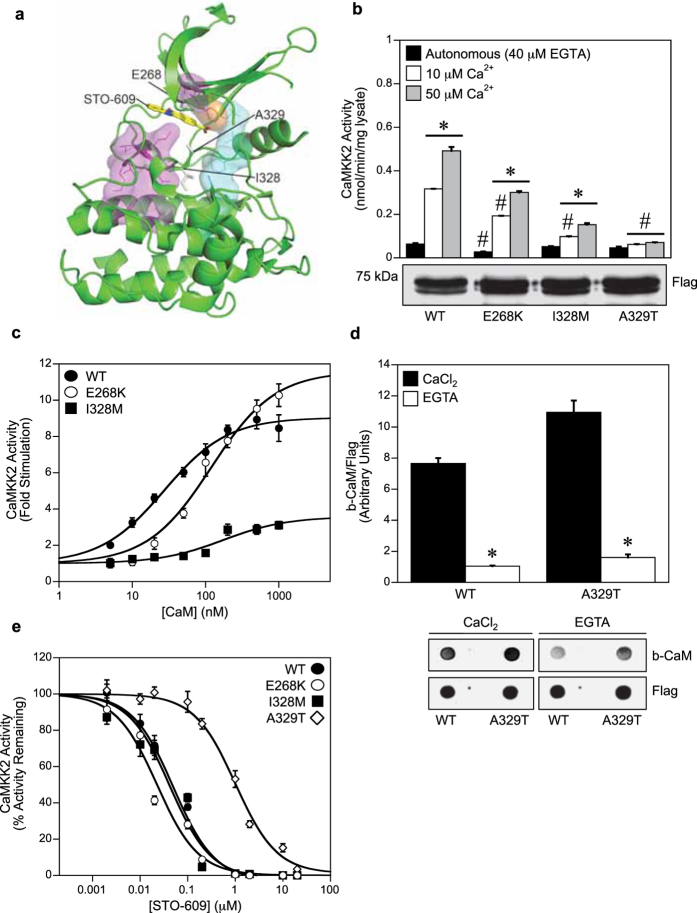
The A329T mutant is insensitive to Ca^2+^-CaM stimulation. (**a**) Structure of the CaMKK2 kinase domain (pdb: 2ZV2), showing the location of the mutants (E268K, I328M, A329T) with respect STO-609 (yellow). Key structural motifs are shown; R-spine (cyan), C-spine (magenta) and the Phe267 gatekeeper residue (orange). (**b**) Autonomous and Ca^2+^-CaM stimulated activities of WT and mutant CaMKK2 determined in the presence of 40 μM EGTA, 1 μM CaM, 200 μM ATP and either 10 μM or 50 μM Ca^2+^. CaMKK2 activity was measured using the CaMKKtide peptide substrate assay and presented as the mean ± SEM; n = 4. The lower panel shows expression levels of WT CaMKK2 and the mutants as determined by immunoblot using a rabbit anti-Flag antibody. (**c**) Kinase activity of WT and mutant CaMKK2 measured over a range of CaM concentrations (0–1000 nM) in the presence of 50 μM Ca^2+^. CaMKK2 activity was measured using the CaMKKtide peptide substrate assay and fitted to the equation: Activity = Basal + ((((Fold Stimulation × Basal) − Basal) × [CaM])/(A_0.5_ + [CaM])), where A_0.5_ is the concentration of CaM giving half-maximal stimulation. Data are presented as mean ± SEM; n = 4. (**d**) Overlay analysis of CaM binding to WT and A329T mutant CaMKK2. Binding of biotinylated CaM and total CaMKK2 was visualised using fluorescently-labeled streptavidin and rabbit anti-Flag antibody, respectively. The bar chart shows b-CaM binding (quantified from the overlay), relative to total CaMKK2 protein. Data are presented as mean ± SEM; n = 3. (**e**) Kinase activity of WT and mutant CaMKK2 measured over a range of STO-609 concentrations (0–20 μM) in the presence of 50 μM Ca^2+^, 1 μM CaM and 200 μM ATP. CaMKK2 activity is expressed as a percentage of the activity measured in the absence of STO-609, then fitted to the equation: Activity = Minimum Activity + ((Maximal Activity − Minimum Activity)/1 + ([STO-609]/IC_50_)), where IC_50_ is the concentration of STO-609 giving half-maximal inhibition. Data are presented as mean ± SEM; n = 4. Statistical analysis was performed by two-way ANOVA. *p < 0.01, vs the control within the same group; ^#^p < 0.01 vs WT within the same treatment.

Three rare-variant point mutations (E268K; I328M, rs149343557; A329T, rs768551666) that occur close to the ATP-binding site of the catalytic domain of human CaMKK2 (Fig. 4a), have been detected in two large-scale genetic studies^15^,^24^. Additionally, the E268K mutation has also been found in the acute lymphoblastic leukemia cell line MOLT-4, whereas the A329T mutation has been detected in tissue samples derived from endometrial carcinoma^18^,^30^. As shown in Fig. 4b, the E268K mutant had significantly reduced autonomous activity compared with WT CaMKK2. The E268K and I328M mutants also exhibited lower Ca^2+^-CaM stimulated activity at both Ca^2+^ concentrations, however the A329T mutant was virtually insensitive to Ca^2+^-CaM stimulation despite similar levels of expression to WT CaMKK2 as shown by immunoblot. The concentration of CaM required to give half-maximal stimulation was also significantly increased for the E268K and I328M mutants, indicating loss of CaM sensitivity (Fig. 4c; Table 1). The I328M mutation also caused a marked reduction in CaM dependence (8.64-fold stimulation for WT versus 3.11-fold for I328M). Since the A329T mutant was insensitive to Ca^2+^-CaM stimulation, we next tested whether this mutation disrupted Ca^2+^-CaM binding. Using CaM overlay analysis, we found that the A329T mutant bound CaM to a comparable extent as WT CaMKK2, and in a Ca^2+^-dependent manner (Fig. 4d).

The small-molecule CaMKK2 inhibitor STO-609 has been shown to regress tumor burden in mouse models of prostate and hepatic cancers^6^,^7^,^8^. The A329T mutation was previously shown to desensitize CaMKK2 to STO-609 inhibition^31^, therefore we tested whether the other mutations have a similar effect. In agreement with the previous study, we found the A329T mutation rendered CaMKK2 more resistant to STO-609 inhibition, however the E268K and I328M mutants were inhibited by STO-609 with half-maximal concentrations similar to WT CaMKK2 (Fig. 4e).

### The G87R and R139W mutations decrease AMPK phosphorylation in response to ionomycin

**Figure 5:**
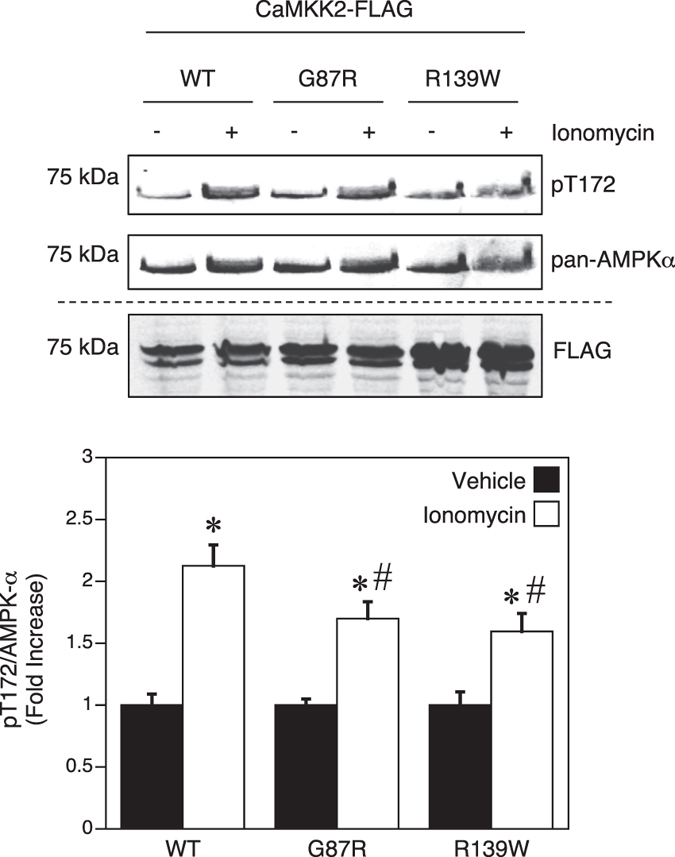
Expression of the G87R and R139W mutants in HeLa cells decreases AMPK Thr172 phosphorylation in response to ionomycin. HeLa cells expressing either recombinant Flag-tagged CaMKK2 WT, G87R or R139W mutants were treated with vehicle (DMSO) or 5 μM ionomycin 5 min prior to lysis. Phosphorylation of Thr172 and total AMPK-α subunit protein was determined (shown on a cropped representative immunoblot) using rabbit pThr172 and mouse AMPK-α antibodies, and visualised simultaneously with goat anti-mouse IgG IRDye800 and goat anti-rabbit IgG IRDye680 fluorescently labeled secondary antibodies on a Infrared Imager. Expression of the Flag-tagged CaMKK2 constructs from the same lysates was determined on a separate immunoblot using rabbit anti-Flag and goat anti-rabbit IgG IRDye680 antibodies. The bar chart shows Thr172 phosphorylation quantified from the immunoblot, expressed as fold increase relative to the vehicle control. Data are presented as mean ± SEM; n = 3. Statistical analysis was performed by two-way ANOVA. *p < 0.01, vs the vehicle control within the same group; ^#^p < 0.05 vs WT treated with ionomycin.

Since the G87R and R139W mutations gave rise to the largest decrease in autonomous and Ca^2+^-CaM stimulated activities among the mutants tested, we examined whether they can behave as dominant-negative inhibitors of CaMKK2 signaling in cells. For these experiments, we over-expressed WT CaMKK2, G87R and R139W mutants in HeLa cells to test whether they interfere with downstream phosphorylation of AMPK on Thr172 in response to ionomycin (a Ca^2+^-ionophore). The advantage of using AMPK phosphorylation as a read out of CaMKK2 signaling is that CaMKK2 is the sole upstream kinase for AMPK Thr172 in HeLa cells^32^. Stimulation with ionomycin resulted in a 2.1-fold increase in Thr172 phosphorylation in HeLa cells over-expressing WT CaMKK2, whereas phosphorylation was significantly reduced in cells over-expressing either the G87R or R139W mutant (38% and 47% reduction, respectively) (Fig. 5).

## Discussion

In this study, we report the effects of nine rare-variant point mutations on the regulation of human CaMKK2 by multi-site phosphorylation, Ca^2+^-CaM and sensitivity to the small-molecule inhibitor STO-609. Rare genetic variants are thought to contribute to complex disease risk, since large genome-wide studies of common variants have explained only a small proportion of the known heritability of most complex disorders^21^. Since aberrant CaMKK2 activity is strongly associated with behavioral disorders and several types of cancer, understanding the functional consequences of these mutations offers new insight into disease risk and burden of disease in human populations carrying these variants.

The G87R mutation significantly reduced CaMKK2 autonomous activity, and rendered the enzyme incapable of further increasing autonomous activity via Thr85 autophosphorylation. This is similar to the effect of the T85S mutation in CaMKK2 that is linked to bipolar and anxiety disorder in humans^4^. The R139W and R142W mutants also exhibit decreased autonomous activity despite having substantially reduced phosphorylation on Ser129 and Ser133. These mutants are therefore unable to increase autonomous activity in response to Ser129/Ser133/Ser137 dephosphorylation and are uncoupled from regulation by the Ser137 priming kinases and GSK3. Increasing autonomous activity via dephosphorylation of these sites is critical for promoting neurite outgrowth and appropriate axon-dendrite polarity in neurons, as a S129D/S133D/S137D phospho-mimetic mutant that is incapable of being dephosphorylated is unable to rescue growth defects in neuronal cells from CaMKK2 null mice, whereas expression of WT CaMKK2 or a S129A/S133A/S137A mutant is able to restore normal morphology^5^. Several studies have reported an association between reduced CaMKK2 activity and schizophrenia in humans^11^,^33^,^34^. Consistent with these findings, we reported previously that CaMKK2-null mice display behavioral disturbances similar to those observed in humans with schizophrenia^4^. Strikingly, the decrease in CaMKK2 autonomous activity in the G87R and R139W mutants (>50% decrease) is similar in magnitude to the reduction in CaMKK2 observed in schizophrenic and bipolar patients^11^,^35^. Since the G87R and R139W mutants exert a dominant-negative effect on CaMKK2 signaling, these mutations could be considered as potential risk markers for these behavioural disorders.

Analysis of the S137L and P138S mutants revealed they lacked phosphorylation on Ser129, but unlike the R139W and R142W mutants, had significantly increased autonomous activity compared with WT CaMKK2. Notably, the autonomous activity of the P138S mutant is comparable in magnitude to the Ca^2+^-CaM stimulated activity of WT CaMKK2 measured at 10 μM Ca^2+^, indicating that the P138S mutation confers gain-of-function and will likely be in an activated state under resting conditions in the cell. Elevated CaMKK2 activity has been shown to play an adverse role in HCC as well as prostate cancer. CaMKK2 is generally increased in tumors from HCC patients compared with adjacent normal tissue, and these elevated levels negatively correlate with HCC patient survival^6^. Moreover, large scale expression and proteomic studies show increased CaMKK2 activity in prostate cancer, and CaMKK2 has been shown to play a role in progression to prostatic malignancy^36^,^37^. Collectively, our findings raise the possibility that human populations harboring the S137L or P138S mutations may have increased risk from developing HCC and/or prostate cancer.

The dichotomy between decreased CaMKK2 activity and schizophrenia, and increased CaMKK2 activity and prostate cancer, is borne out by an inverse co-morbidity between the two diseases^38^. It has been reported that male schizophrenic patients have a lower risk of prostate cancer^39^, and meta-analysis of cancer incidence of immediate family relatives of schizophrenic patients found a significantly lower risk of cancer among their unaffected parents and siblings^40^,^41^. These findings indicate that a genetic predisposition towards schizophrenia may reduce susceptibility to some cancers, and it is possible that certain CaMKK2 mutations contribute to this phenomenon.

Of the three mutations that occur in the catalytic domain, the E268K and A329T mutants had the greatest effect on CaMKK2 activity and Ca^2+^-CaM regulation. The E268K mutant had significantly lower autonomous and Ca^2+^-CaM stimulated activity, similar to the effect of the G87R and R139W mutants, and could be considered a risk marker for disorders associated with decreased CaMKK2 activity. Consistent with previous studies^31^, we found that the A329T mutation made CaMKK2 more resistant to STO-609 inhibition. The A329T mutation has been detected in endometrial carcinoma and is analogous to the A380T mutation that occurs in the oncogenic Bcr-Abl fusion, which was shown to cause imatinib resistance in patients with chronic myeloid leukemia^42^. This has implications for future development of therapeutic inhibitors of CaMKK2 that target the ATP-binding site, which should take into account potential drug resistance that may arise from mutations at the Ala329 position. As well as desensitizing CaMKK2 to STO-609 inhibition, we found that the A329T mutation also rendered CaMKK2 insensitive to Ca^2+^-CaM stimulation despite being able to bind CaM. Ala329 is located immediately N-terminal to the DFG motif at the start of the activation loop, but is likely to be distally located from the CaM binding site based on the crystal structures of other CaMK family members (CaMKII, CaMKIV and DAPK1)^43^,^44^,^45^. Binding of Ca^2+^-CaM is thought to stimulate CaMKK2 activity by sequestering the autoinhibitory sequence from the active site^3^, however its possible that a secondary rearrangement of the activation loop is also required, and that this conformational change is not permitted in the A329T mutant.

In summary, we propose that several of the rare-variant CaMKK2 mutations (particularly the G87R, S137L, P138S, R139W, E268K and A329T mutants) have a functional basis for being risk markers for susceptibility to diseases associated with aberrant CaMKK2 activity. As these mutations are rare with a global allele frequency of <1%, this may indicate that there is evolutionary pressure on the coding region of CaMKK2, particularly around these important regulatory sites, to prevent accumulation of deleterious variations within the human population.

## Methods

### Molecular biology

A plasmid construct for full length C-terminal Flag-tagged human CaMKK2 isoform-1 was cloned into pcDNA3(−) using XhoI/HindIII restriction sites for expression in COS7 cells. Point mutations were generated using Quikchange site-directed mutagenesis (Stratagene), and all plasmid constructs were verified by sequencing the entire open reading frame. DNA for transfection was prepared using Wizard *Plus* SV Miniprep DNA Purification Kits (Promega) and quantified by absorbance at 260 nm.

### Expression of recombinant CaMKK2 and mutant variants

COS7 cells were grown in DMEM (Sigma) media with 10% fetal calf serum at 37 °C with 5% CO_2_. Cells were transfected at 60% confluency with 2 μg of pcDNA3 containing C-terminal Flag-tagged human or various point mutants using FuGene HD (Roche). CaMKK2 expressed under standard culturing conditions exists primarily as a triply phosphorylated species, which we showed previously is due to constitutive phosphorylation of Ser129/Ser133/Ser137^5^. The triply phosphorylated CaMKK2 species is fully Ca^2+^-CaM dependent, and can readily undergo Thr85 autophosphorylation in the presence of Ca^2+^-CaM and MgATP. Transfected cells were harvested after 48 hr by washing with ice-cold phosphate-buffered saline (PBS) followed by rapid lysis *in situ* using 1 ml of lysis buffer (50 mM Tris.HCl [pH 7.4], 150 mM NaCl, 50 mM NaF, 1 mM NaPPi, 1 mM EDTA, 1 mM EGTA, 1 mM DTT, 1% [v/v] Triton X-100) containing Complete protease inhibitor cocktail (Roche). Cellular debris was removed by centrifugation and total protein was determined using the Bradford protein assay (Pierce). Recombinant CaMKK2 was purified from 2 μg of cell lysate using 10 μl of anti-Flag M2 agarose (50% v/v) (Sigma) pre-equilibrated in lysis buffer, followed by successive washes in lysis buffer containing 1 M NaCl, and finally into 50 mM HEPES [pH 7.4]. The immobilized CaMKK2 was then sedimented by centrifugation and used for kinase assays, or the CaMKK2 was eluted from the beads by incubating overnight at 4 °C with two bead volumes of Flag peptide (1 mg/ml), and used for the CaM overlay and MS/MS phosphopeptide analysis.

### CaMKK2 activity assay

CaMKK2 activity was measured using a synthetic peptide substrate (CaMKKtide) as previously described^4^. For a standard 30 μl assay, 10 μl of recombinant CaMKK2 immobilized on anti-Flag M2 agarose beads (50% v/v) was incubated in assay buffer (50 mM HEPES [pH 7.4], 1 mM DTT, 0.02% [v/v] Brij-35) containing 200 μM CaMKKtide, 10 or 50 μM CaCl_2_, 1 μM CaM (Sigma), 40 μM EGTA, 200 μM [γ-^32^P]-ATP (Perkin Elmer) and 5 mM MgCl_2_. All the kinase reactions were buffered with 40 μM EGTA, which we determined by titration to chelate trace amounts of Ca^2+^ present in the CaM preparations. This was essential to measure the stimulation of CaMKK2 activity at the 10 μM Ca^2+^ concentration^46^. Reactions were incubated at 30 °C for 10 min, after which they were terminated by spotting 15 μl onto P81 phosphocellulose paper and washing extensively in 1% phosphoric acid^47^. Radioactivity was quantified by liquid scintillation counting. CaMKK2 activity is presented as specific activity (nmol/min/mg lysate) and was corrected for minor differences in CaMKK2 expression as determined immunoblotting using an anti-Flag antibody and Odyssey Infrared Imager (See below). For autophosphorylation reactions, the first step was performed using 50 μl of anti-Flag agarose immobilized CaMKK2 that was incubated with 5 mM MgCl_2_, 50 μM CaCl_2_ and 1 μM CaM in the presence and absence of 200 μM ATP (Sigma). Reactions were incubated at 30 °C for various times, after which the beads were washed successively in lysis buffer containing 1 M NaCl, and finally re-suspended in 50 mM HEPES [pH 7.4] to achieve a 50% slurry. A 10 μl aliquot (50% slurry) was removed and kinase activity measured in assay buffer containing 200 μM CaMKKtide, 50 μM CaCl_2_, 1 μM CaM, 200 μM [γ-^32^P]-ATP and 5 mM MgCl_2_, in the presence or absence of 1 mM EGTA using the CaMKKtide peptide substrate assay described above. Based on CaMKK2 having a specific activity of 1 μmol.min^−1^.mg^−1^ the concentration of CaMKK2 in the pull down assays is approximately 0.5 nM.

### Immunoblotting

Cell lysates or immuno-precipitated CaMKK2 were denatured in SDS sample buffer, separated by SDS-PAGE and transferred to Immobilon PVDF membranes (Millipore). Membranes were blocked for 1 hr in PBS/1% Tween-20 (PBS-T) supplemented with 2% non-fat milk. Primary antibodies were diluted in PBS-T containing 1% non-fat milk at the following dilution: rabbit or mouse anti-Flag (Cell Signaling; Cat No 2368 S; Lot No 6; 100 ng/ml or Cat No 8146 S; Lot No 3; 100 ng/ml, respectively), rabbit anti-pThr85 (300 ng/ml)^4^, rabbit anti-pThr172 (Cell Signaling; Cat No 2535 L; Lot No 16; 100 ng/ml), mouse anti-pan AMPK-α (Cell Signaling; Cat No 2793 S; Lot No 6; 100 ng/ml) and mouse anti-pSer antibody (BD Bioscience; Cat No 612546; Lot No 4232670; 250 ng/ml), which selectively detects phosphorylated Ser129 on CaMKK2^5^. After incubation with primary antibody solutions for 1 hr, the membranes were briefly washed in PBS-T, and then incubated with appropriate fluorescently labeled secondary antibodies, either goat anti-mouse IgG IRDye800 (Li-Cor) or goat anti-rabbit IgG IRDye680 (Li-Cor) for 1 hr. After successive washing with PBS-T, the membranes were then scanned with an Odyssey Infrared Imager (Li-Cor).

### CaM overlay

1 μg of purified WT and A329T mutant CaMKK2 were spotted onto nitrocellulose membrane, which was then blocked for 1 hr in PBS/1% Tween-20 (PBS-T) supplemented with 2% non-fat milk. The membrane was incubated overnight at 4 °C with biotinylated CaM (500 nM; Millipore) diluted in PBS-T containing 1% non-fat milk and 10 mM CaCl_2_. The membranes were briefly washed in PBS-T containing 10 mM CaCl_2_, and then incubated with IR680 dye labeled streptavidin (Li-Cor) for 1 hr. After successive washing with PBS-T containing 10 mM CaCl_2_, the membranes were then scanned with an Odyssey Infrared Imager. The membranes were then incubated in PBS-T containing 15 mM EGTA overnight at 4 °C in order to determine if the biotinylated CaM bound in a Ca^2+^ dependent manner.

### Mass spectrometry

In order to analyse Thr85 autophosphorylation in the G87R mutant, 1 μg of WT CaMKK2 or G87R mutant was incubated with 5 mM MgCl_2_, 50 μM CaCl_2_ and 1 μM CaM in the presence and absence of 200 μM ATP for 40 min, then digested with trypsin and analyzed by reversed-phase HPLC-ESI-MS/MS using an UltiMate 3000 Nano LC HPLC system (Dionex) directly connected to a Triple-TOF 5600 mass spectrometer (AB SCIEX) in direct injection mode. Peptide mixtures were resolved on an analytical nanocapillary HPLC column (100 μm i.d. × 15 cm) packed with C_18_ Acclaim PepMap100 (3 μm particle size, 100 Å pore size) using a 1–75% elution gradient of 98% acetonitrile/2% of 0.1% formic acid (v/v) in water at a flow rate of 250 nl/min. Mass spectrometric data were analyzed using the database search engine ProteinPilot and the Paragon algorithm (AB Sciex).

### Statistical analysis

Results are expressed as the mean ± standard error of mean (SEM). Statistical analysis, where indicated, was performed using two-way analysis of variance (ANOVA), with the alpha level set at 0.05 for each test. P-values of less than 0.05 were considered statistically significant.

## Additional Information

**How to cite this article**: O’Brien, M. T. *et al*. Impact of Genetic Variation on Human CaMKK2 Regulation by Ca^2+^-Calmodulin and Multisite Phosphorylation. *Sci. Rep.* **7**, 43264; doi: 10.1038/srep43264 (2017).

**Publisher's note:** Springer Nature remains neutral with regard to jurisdictional claims in published maps and institutional affiliations.

## Supplementary Material

Supplementary Information

## Figures and Tables

**Table 1:** Effect of the rare-variant mutations on CaM sensitivity and dependence.

CaMKK2 mutation	Fold stimulation in the presence of Ca^2+^-CaM (relative to activity in the absence of CaM)	Concentration of CaM required for half-maximal stimulation (nM)
WT	8.6 ± 0.3	29.5 ± 2.5
G87R	13.2 ± 0.2*	20.7 ± 3.7
R91W	9.8 ± 1.0	33.4 ± 11.8
S137L	5.0 ± 0.3*	22.4 ± 7.6
P138S	5.4 ± 0.2*	14.8 ± 3.4*
R139W	10.6 ± 0.8	16.7 ± 6.6*
R142W	18.1 ± 0.6*	32.3 ± 4.9
E268K	11.5 ± 0.6	119 ± 20.1*
I328M	3.8 ± 0.3*	173 ± 69.0*

The Ca^2+^-CaM stimulated activities of WT and mutant CaMKK2 measured over a range of CaM concentrations (0–1000 nM) in the presence of a fixed concentration of Ca^2+^ (50 μM), expressed relative to activity in the absence of CaM. Data are presented as mean ± SEM; n = 4. Statistical analysis was performed by two-way ANOVA. *p < 0.01, vs WT.
