# Supplementary material for: Impact of Genetic Variation on Human CaMKK2 Regulation by Ca2+-Calmodulin and Multisite Phosphorylation
Source: Sci Rep. 2017 Feb 23;7:43264. doi: 10.1038/srep43264 (PMC5322397; doi:10.1038/srep43264)

SUPPLEMENTARY INFORMATION

Impact of Genetic Variation on Human CaMKK2 Regulation by Ca^{2+}-Calmodulin and Multisite Phosphorylation

Matthew T. O'Brien, Jonathan S. Oakhill, Naomi X. Y. Ling, Christopher G. Langendorf, Ashfaqul Hoque, Toby A. Dite, Anthony R. Means, Bruce E. Kemp and John W. Scott

SUPPLEMENTARY FIGURES

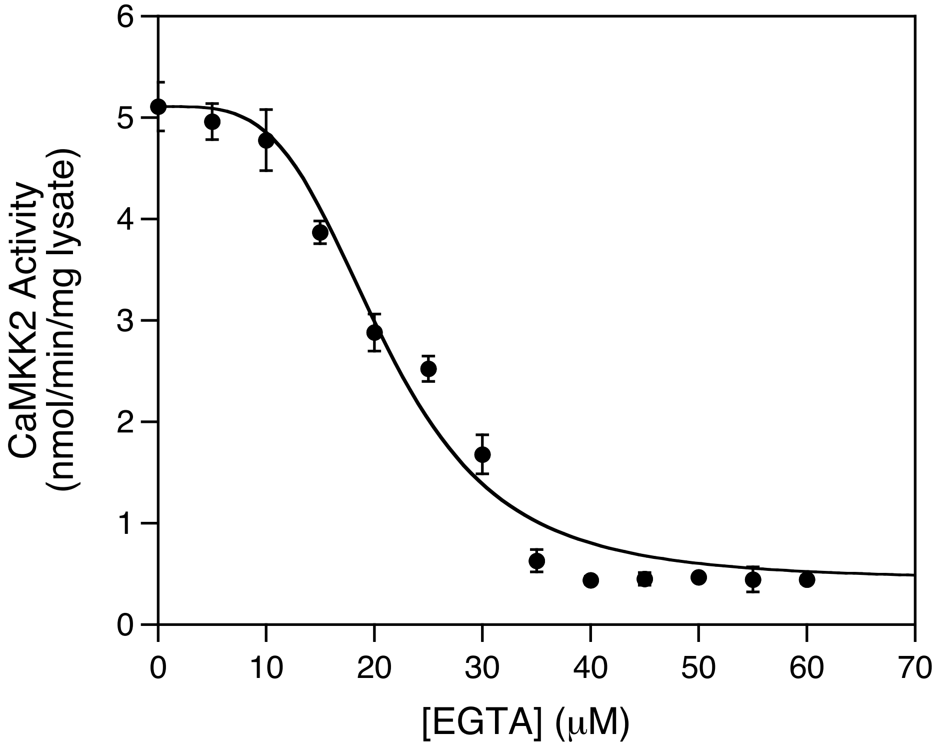

Supplementary Figure 1: CaMKK2 activity measured in the presence of CaM and increasing concentrations of EGTA.

CaMKK2 activity was measured in the presence of 200 μM ATP, 1 μM CaM and increasing concentrations of EGTA, in order to determine the EGTA concentration required to chelate contaminating Ca^{2+} in the CaM preparation for measuring Ca^{2+}-independent autonomous activity. CaMKK2 activity was measured using the CaMKKtide peptide substrate assay and data were fitted to the equation: Activity = Minimum Activity + ((Maximal Activity - Minimum Activity)/1 + $([EGTA]/IC_{50})^h$). Data are presented as mean ± SEM; n=3.

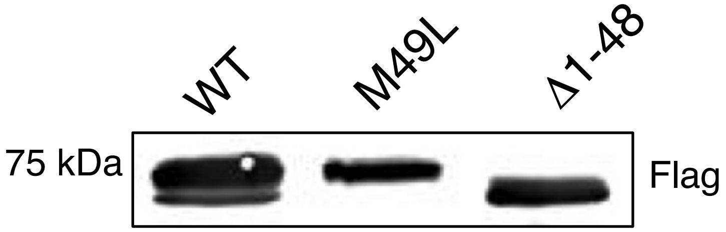

Supplementary Figure 2: CaMKK2 undergoes alternative translation initiation from Met49. Immunoblot analysis of WT CaMKK2, as well as a M49L point mutant and Δ1-48 deletion mutant. The proteins were visualised using a rabbit anti-Flag antibody, and goat anti-rabbit IgG IRDye680 fluorescently labeled secondary antibody on a Infrared Imager.

Supplementary Figure 3: Phosphorylation profile of full length CaMKK2 and the shorter alternative Met49 initiated translation species.

De-convoluted whole-protein TOF spectra of recombinant Flag-tagged human CaMKK2 expressed in COS7 cells under standard culture conditions (DMEM +10% FBS). (**a**) TOF spectra of full length CaMKK2 (upper band). (**b**) TOF spectra of the alternative Met49 initiated translation species (lower band). P denotes the number of phosphate modifications.

a

CaMKK2 (Upper band)

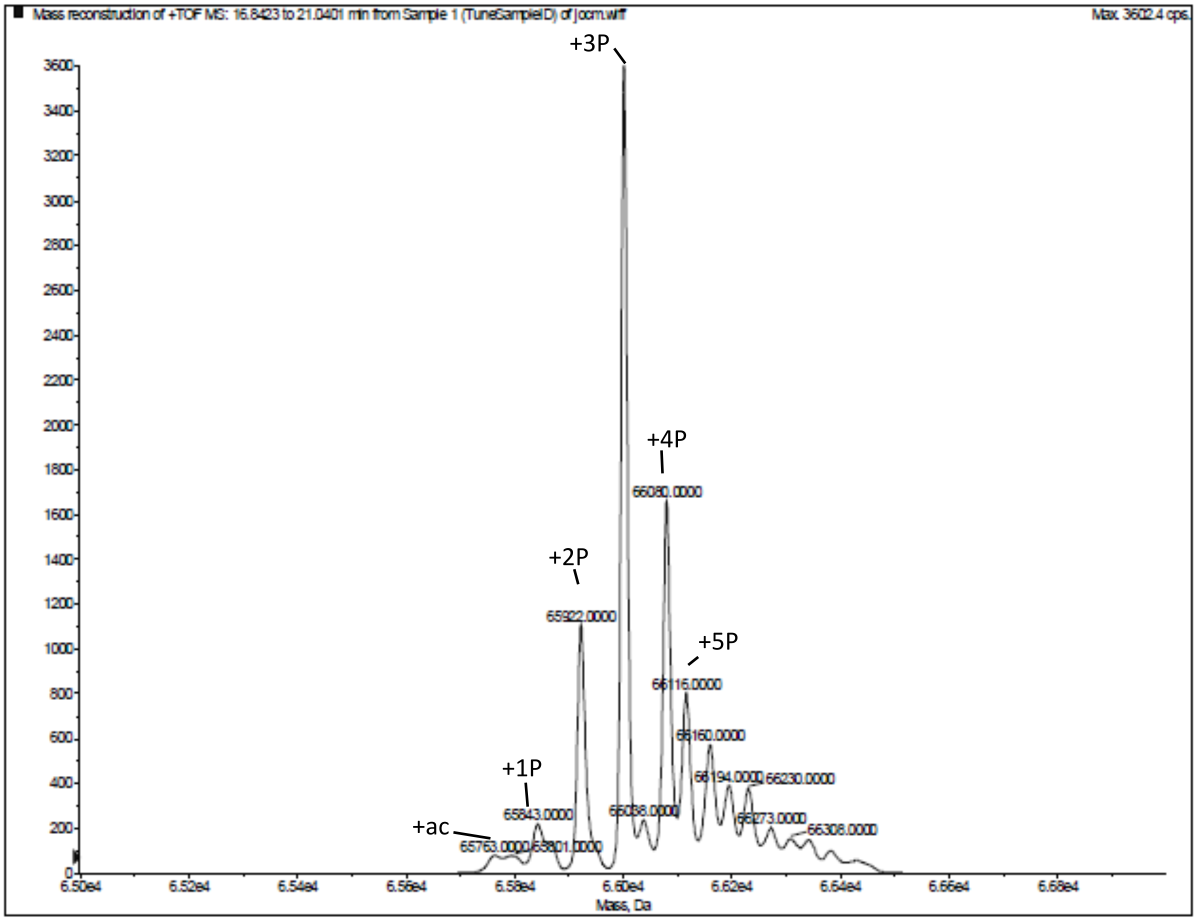

b

CaMKK2 (Lower band)

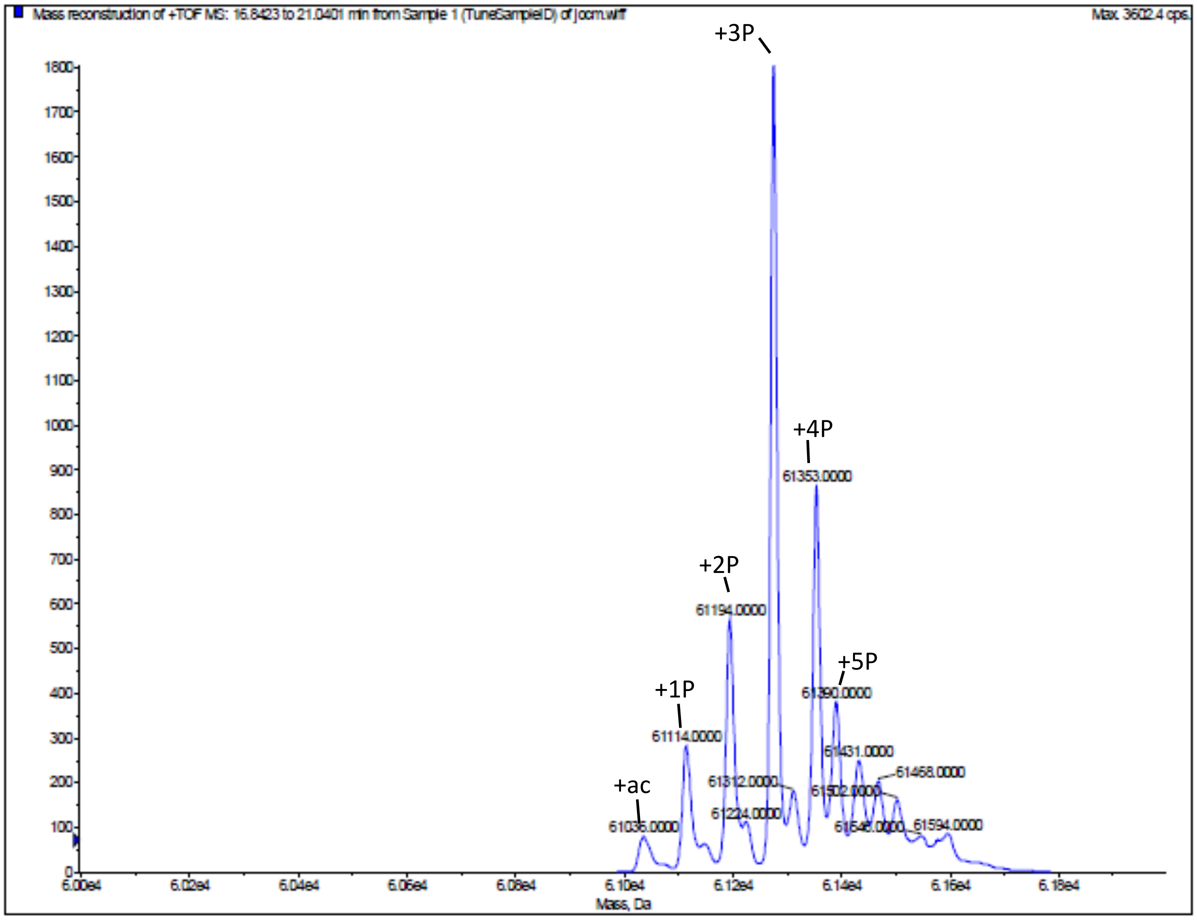

Supplementary Figure 4: The G87R mutation blocks Thr85 autophosphorylation.

ESI-tandem mass spectrometry analysis of Thr85 autophosphorylation in WT and G87R mutant CaMKK2. The representative extracted ion chromatograms in each panel show the proportion of Thr85-phosphorylated peptide (pThr85) to non-phosphorylated peptide (Thr85) from a tryptic digest of wild-type (upper panels) and G87R mutant CaMKK2 (lower panels), determined at 0 min (Control) and 40 min after incubation with 50 μM Ca^{2+}, 1 μM CaM and 200 μM MgATP (Autophosphorylation).

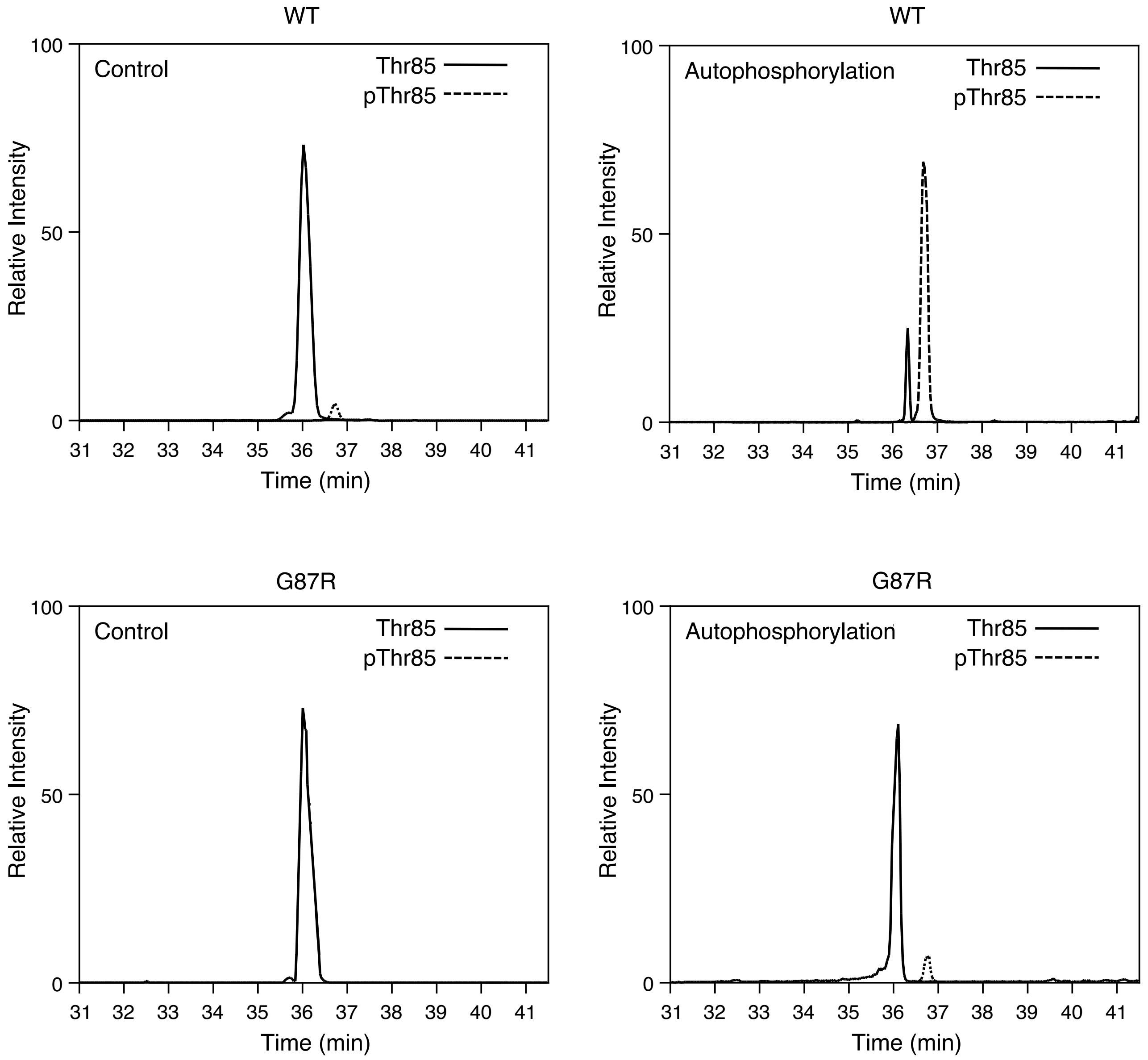

Supplement: Supplementary Information [file srep43264-s1.pdf]
